# The Association of Cell-Free LncH19 and miR-29b Expression with the PI3K/AKT/HIF-1/VEGF Pathway in Patients with Diabetic Nephropathy: In Silico Prediction and Clinical Validation

**DOI:** 10.3390/cimb47010020

**Published:** 2024-12-31

**Authors:** Noha M. Abd El-Fadeal, Basma Osman Sultan, Asmaa K. K. AbdelMaogood, Essam Al Ageeli, Fatma Tohamy Mekhamer, Sherihan Rohayem, Ahmed Shahidy, Nora Hosny, Manal S. Fawzy, Mohammed M. Ismail, Hidi A. A. Abdellatif

**Affiliations:** 1 Medical Biochemistry and Molecular Biology Department, Faculty of Medicine, Suez Canal University, Ismailia 41522, Egypt; noha_abdelfadeal@med.suez.edu.eg (N.M.A.E.-F.); or nora_hosny@med.suez.edu.eg (N.H.); haidy.azmy@med.suez.edu.eg (H.A.A.A.); 2 Department of Biochemistry, Ibn Sina National College for Medical Studies, Jeddah 22421, Saudi Arabia; 3 Oncology Diagnostic Unit, Faculty of Medicine, Suez Canal University, Ismailia 41522, Egypt; 4 Nephrology Unit, Internal Medicine Department, Faculty of Medicine, Suez Canal University, Ismailia 41522, Egypt; basma.osman@med.suez.edu.eg; 5 Clinical and Chemical Pathology Department, Faculty of Medicine, Suez Canal University, Ismailia 41522, Egypt; asmaa.kamal@med.suez.edu.eg; 6 Department of Basic Medical Sciences, Faculty of Medicine, Jazan University, Jazan 45141, Saudi Arabia; ealageeli@jazanu.edu.sa; 7 Family Medicine Department, Faculty of Medicine, Suez Canal University, Ismailia 41522, Egypt; fatma.tohamy@med.suez.edu.eg; 8 Microbiology and Medical Immunology Department, Faculty of Medicine, Port Said University, Port Said 42523, Egypt; 9 Cardiothoracic Surgery Resident, Faculty of Medicine, Suez Canal University, Ismailia 41522, Egypt; ahmedelshahidy44@icloud.com; 10 Center of Excellence in Molecular and Cellular Medicine, Faculty of Medicine, Suez Canal University, Ismailia 41522, Egypt; 11 Center for Health Research, Northern Border University, Arar 91431, Saudi Arabia; 12 Department of Anatomy, Faculty of Medicine, Northern Border University, Arar 91431, Saudi Arabia; 2351357609@nbu.edu.sa; 13 Medical Biochemistry and Molecular Biology Department, Faculty of Medicine, King Salman International University, Tur Sinai 46618, Egypt

**Keywords:** diabetic nephropathy, lncRNA H19, miRNA29b, PI3K, AKT, HIF-1 alpha

## Abstract

Diabetic nephropathy (DN) affects about one-third of patients with diabetes and can lead to end-stage renal disease despite numerous trials aimed at improving diabetic management. Non-coding RNAs (ncRNAs) represent a new frontier in DN research, as increasing evidence suggests their involvement in the occurrence and progression of DN. A growing body of evidence suggests that long non-coding RNAs (lncRNAs) and microRNAs (miRNAs) in DN signaling pathways might serve as novel biomarkers or therapeutic targets, although this remains to be fully explored. Our study included four groups, each comprising 40 adults: patients with diabetes (a) without albuminuria, (b) with microalbuminuria, (c) with macroalbuminuria, and a control group. All participants underwent history-taking and clinicolaboratory assessments, including CBC, fasting blood sugar, HbA1c, lipid profile, liver function, and renal function tests. Additionally, expressions of lncRNA H19, miRNA-29b, PI3K, AKT, mTOR, and HIF-1 alpha were assessed using qPCR. lncRNA H19 expression was upregulated in patients with albuminuria compared to the DM group. Furthermore, based on qPCR, the level of lncRNA H19 was negatively correlated with eGFR and miRNA-29b expression. On the other hand, the lncRNA H19 level was positively correlated with PI3K, AKT, mTOR, and HIF-1 alpha levels. We also found that the lncH19/miRNA-29b ratio was significantly increased in patients with DN and macroalbuminuria. In conclusion, lncRNA H19 was upregulated in patients with DN, and this increase was associated with miRNA29b downregulation. Therefore, our study suggests a novel link between the lncH19/miRNA-29b ratio and DN, indicating that it might serve as a potential biomarker for the dynamic monitoring of DN.

## 1. Introduction

Diabetic nephropathy (DN) is a major contributor to end-stage renal disease (ESRD) that is associated with increased morbidity and mortality. The “World Health Organization” estimates that by 2030, approximately 370 million people will live with diabetes mellitus (DM) [1]. Patients with DM are at risk of progressing to nephropathy, as DN affects nearly one-third of those individuals. Among diabetic complications such as retinopathy, neuropathy, and macrovascular changes, DN is the leading cause of chronic kidney disease and ESRD in these patients [2,3]. Therefore, developing effective therapeutic targets to halt the progression of these complications and reduce associated risks is essential [4].

Recent evidence highlights the roles of hypoxia and oxidative stress in DN development, suggesting that non-coding RNAs (ncRNAs) could open new treatment avenues for diabetic kidney diseases [5]. Long non-coding RNAs (lncRNAs), in particular, play vital roles in multiple cellular processes, including transcriptional and post-transcriptional regulation, as well as chromatin organization. LncRNAs can regulate gene expression through various mechanisms, including the repression of translation and the enhancement of gene expression. This regulatory capacity plays a critical role in disease modulation [6]. A promising aspect of lncRNAs is their greater specificity toward particular tissues or cell types compared to mRNAs or miRNAs, indicating their potential as superior biomarkers [7].

LncRNAs have been recognized as essential molecules that play a significant role in the pathophysiology of renal diseases, and they may serve as valuable biomarkers for the early diagnosis and prognosis of individuals with kidney diseases [7]. One such lncRNA, H19, located on chromosome 11p15.5, is transcribed alongside the insulin-like growth factor-2 from a conserved gene cluster [8]. During kidney development, lncH19 expression increases in ureteric bud branches and nephron epithelium, but decreases after birth. Reactivation and elevated H19 expression are linked to tumor activation, tissue proliferation, DNA repair, and increased cellular stress. Numerous studies highlight the significance of H19 in aging, cancer, and conditions associated with increased cellular proliferation [8,9].

Subcellular localization studies show that lncH19 is expressed in the nucleus, cytoplasm, mitochondria, and endoplasmic reticulum, where it regulates transcriptional factors or acts as an enhancer or suppressor of transcription. It also influences alternative splicing and many post-transcriptional modifications by forming specific lncRNA-protein complexes, thus impacting the translational process [10]. It has been found that lncRNA H19 exhibits elevated expression levels under hyperglycemic conditions and plays a crucial role in various pathophysiological mechanisms of DN [11].

MicroRNAs (miRNAs) are short RNA sequences of about twenty-two nucleotides long and play vital roles in regulating protein-coding gene expressions. Many miRNAs affect diabetes development and glucose regulation [12,13,14]. The miRNA 29 family, with three members on chromosome seven (i.e., miR-29a, miR-29b, and miR-29c), shows changes in expression levels in diabetic rodent models, especially in kidney and adipose tissues [15,16].

Previous data and bioinformatics analyses suggest the sponging of miR-29b by several lncRNAs, including the lncH19, potentially impacting its expression [6]. The “phosphoinositide 3 kinase (PI3K)\mammalian target of rapamycin (mTOR)\hypoxia-inducible factor-1α (HIF)-1α\vascular endothelial growth factor (VEGF)” signaling pathway, critically involved in cellular responses to hypoxia and oxidative stress conditions prevalent in DN [17], has been experimentally proven as one of the miR-29 target pathways [18]. This study aims to explore the relationship between lncH19 and miR-29b expression and the PI3K/AKT/HIF-1/VEGF pathway in DN. By investigating these interactions, we seek to uncover potential molecular mechanisms driving DN progression and identify novel therapeutic targets for preventing or mitigating DN advancement in diabetic patients.

## 2. Materials and Methods

### 2.1. Ethical Statement

Approval from the “Research Ethics Committee at the Faculty of Medicine, Suez Canal University”, identified by code number 5251#, was secured and rigorously followed throughout the study. Participants were allowed to discontinue their involvement in the study at any time. Before data and sample collection, written informed consent was obtained from all participants.

### 2.2. Study Participants

In this preliminary case–control study, 120 patients with DM and 40 age- and sex-matched controls were included. Blood and early-morning urine samples were collected from all participants on the day of data collection.

Patients with DM were recruited from the “Nephrology Inpatient Department at Blinded University Hospitals” and the “Outpatient Diabetic Clinics in Suez Canal University Hospitals” between July 2023 and December 2023. Patients were classified into three groups based on their rate of urinary albumin excretion (UAE): (1) a normoalbuminuria group with UAE rate  <  30 μg/mg; (2) a microalbuminuria group with at least two of three consecutive urine samples having a UAE of 30–300 μg/mg; and (3) a macroalbuminuria group with UAE  >  300 μg/mg [19]. Patients with renal diseases other than diabetic nephropathy, active urinary tract infection, neoplastic disorders, active or chronic infections or inflammatory disorders, severe liver disease, hematological diseases, pregnancy, prior acute myocardial infarction, or occlusive peripheral vascular disease were excluded from the study.

Additional medical data were obtained from the medical records. Blood pressure and body weight/height were measured, and body mass index (BMI) was calculated. Control subjects were randomly chosen and excluded if they had any chronic diseases or were taking regular medication for any reason.

### 2.3. Biochemical and Molecular Analysis

Following a ten-hour fasting period, venous blood samples (8 mL) were collected: 5 mL was added to a plain tube for serum separation, which was carried out by centrifugation at 2500 rpm for 15 min. Three mL was added to an EDTA tube for glycated hemoglobin (HbA1c) measurement and molecular analyses (Cobas Integra, Roche Diagnostics, USA). Routine laboratory measurements were performed, including fasting blood sugar (FBS), blood urea nitrogen (BUN), serum creatinine, and lipid profile (total cholesterol (TC), high-density lipoprotein cholesterol (HDL-c), and triacylglycerol (TG)) via commercially available kits on the Cobas Integra 400 plus Biochemical analyzer (Roche Diagnostics). Low-density lipoprotein cholesterol (LDL-c) concentration was calculated by the Friedewald equation [20].

Clean-catch midstream urine samples (approximately 20 mL) were collected in a sterile plastic tube. These samples were centrifuged at 3000 rpm at 4 °C for ten minutes to eliminate cell debris and particulate matter. The supernatant was aliquoted and stored at −80 °C to avoid multiple freeze–thaw cycles. Urinary albumin and creatinine concentrations were measured using equipment from Siemens Healthcare Diagnostics Inc. (New York, NY, USA), and the results were expressed as the urinary albumin/creatinine ratio (UACR).

Using the “four-variable Modification of Diet in Renal Disease (MDRD)” formula, the estimated glomerular filtration rate (eGFR) was computed, considering the serum creatinine levels, age, sex, and race. The specific calculation formula used was eGFR = 186.3 × (serum creatinine) − 1.154 × (age) − 0.203 × (0.741 if female) [21]. The quantitative estimation of serum hypersensitive C-reactive protein (hs-CRP) was carried out using the BN System by Dade Behring (USA) through particle-enhanced immunonephelometry (Dade Behring, USA).

### 2.4. LncRNA-miRNA-mRNA Enrichment Bioinformatics Analysis

#### 2.4.1. Selection of miRNA

The human microRNA Disease Database was used to find microRNA (miRNA) associated with Diabetes and its complications [22]. This database was accessed using the link: https://www.cuilab.cn/hmdd (last accessed 1 April 2024).

#### 2.4.2. MiRNA and lncRNA Target Prediction

The starBase v2. database, http://starbase.sysu.edu.cn/ (last accessed 2 January 2024) was used to determine the conserved binding sites for miRNA29b with lncRNAs. This database depends on several curated reference resources.

#### 2.4.3. Target Gene Prediction Using In Silico Approach

**Figure 1:**
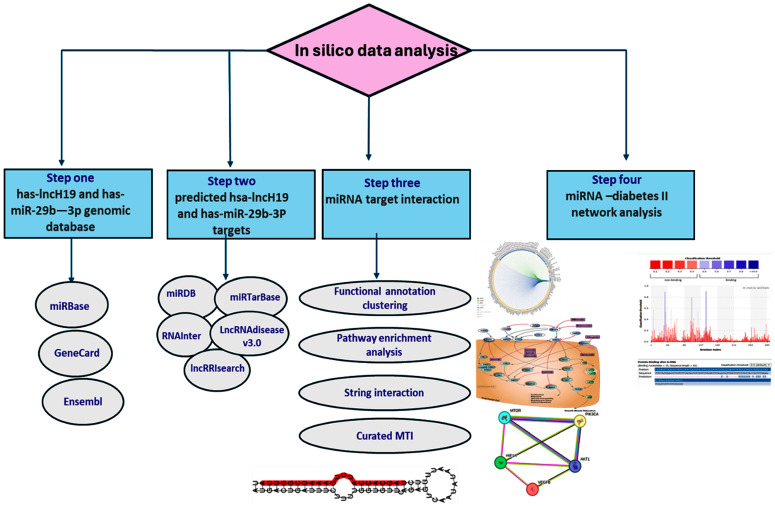
A flow chart of in silico data analysis for the miRNA target prediction enrichment pathway analysis and miRNA–disease interaction.

The in silico data analysis workflow is depicted in Figure 1. Several computational prediction tools were utilized to identify the target genes for both miRNA29b and lncH19. They included miRBase (https://www.mirbase.org/) and miRDB (https://mirdb.org/), which were last accessed 2 January 2024. An intersection of the results and statistical validation/filtration were applied to reduce the false-positive prediction rate.

#### 2.4.4. LncRNA 19 and microRNA Sequence Analysis

The Ensembl and GeneCards Human databases were used to analyze the genetic sequences of miRNA-29b and lncRNA19, as well as to study their functional annotation and disease implications. The databases were accessed via http://www.ensembl.org and https://www.genecards.org, respectively. Both sets of data were last accessed on 22 April 2024.

#### 2.4.5. lncH19, miR-29b, and VEGFA Prediction Binding Analysis

MicroRNA and non-coding RNA predicted protein interaction analysis using the RNAInter v3.0 database available at http://www.rnainter.org (last accessed 2 May 2024). This analysis revealed the top 100 interactors with miRNA and their targets, including lncH19.

#### 2.4.6. Functional Annotation Clustering and Pathway Enrichment Analysis

The predicted miRNA target genes were identified using miRDB, accessed through https://mirdb.org/cgi-bin/search.cgi. Genes were obtained through gene ontology (GO; https://geneontology.org/) and the PANTHER server (https://pantherdb.org/). After target gene prediction, functional annotation clustering and enrichment analyses of the target genes were performed using “Kyoto Encyclopedia of Genes and Genomes (KEGG)” pathways [23], accessed through https://www.genome.jp/kegg/. The strength of interaction between these genes was computed using the STRING database [24], accessed through https://string-db.org/. All mentioned databases were last accessed on 2 January 2024.

### 2.5. Molecular Study

#### 2.5.1. LncH19 and miR-29b Expression Profiling

Total RNA and small RNA were extracted from plasma utilizing the Qiagen miRNeasy Plasma Kit (Cat. No. 74104, Qiagen, Hilden, Germany), following the manufacturer’s protocol. The concentration and purity of the extracted total RNA were assessed by measuring the absorbance ratio at 260/280 nm with the NanoDrop spectrophotometer ND-1000 (NanoDrop Tech., Inc., Wilmington, DE, USA). Subsequently, the total RNA underwent reverse transcription (RT) using the miScript II RT Kit (Qiagen, Catalog no. 218161), which enabled the synthesis of complementary DNA (cDNA) from RNA containing miRNA. This process involved the polyadenylation of miRNAs and other non-coding RNAs using the poly(A) polymerase enzyme, followed by reverse transcription with oligo-dT priming. The reverse transcription occurred in a Veriti™ 96-Well Thermal Cycler (Applied Biosystems, Waltham, MA, USA) at 37 °C for 1 h, followed by the deactivation of the reaction with a short incubation at 95 °C.

SYBR Green kits were utilized to run the real-time polymerase chain reaction (PCR) to examine the expression profiling of lncRNA-miRNA-mRNA. The premixed cDNA was used as a real-time PCR template to measure the expression levels of lncRNA, miRNA, and mRNA. Primers for lncRNA H19, miR-29b, and mRNA (PI3K, AKT, mTOR, and HIF-1alpha) were custom-designed according to the specifications provided by OriGene™ Technologies (https://www.origene.com) (accessed on 10 March 2023) (Table 1). The expression levels were measured using the miScript SYBR Green PCR Kit (Qiagen, Cat. No. 218076). *GAPDH* and U6 small nuclear RNA (snRNA) were used as endogenous controls for data analysis using the ΔΔCT relative quantification method [25,26,27]. The authors carefully considered the selection of these reference genes and confirmed their stability across all four groups in the current analysis. Their expressions remained consistent with minimal variation (i.e., the coefficient of variation for these two reference genes was <5% across all groups), indicating high stability.

Each run included “no-template” and “no-reverse-transcribed” controls, and all reactions were performed in triplicate. The PCR process began with an initial denaturation step at 95 °C for 5 min, followed by 40 cycles at 95 °C (15 s), 60 °C (1 min), and 72 °C (1 min) for denaturation, annealing, and elongation, respectively.

The Livak method was followed to calculate the fold changes for all circulating study biomarkers in each sample relative to the controls based on the quantification cycle (Cq or CT) value, where relative expression = 2^−ΔΔCq^. The expression levels were measured according to the minimal information required for publication guidelines from the quantitative real-time PCR experiment. The application of this method has several key aspects: (1) the method is easy to perform and understand, making it accessible for researchers across various fields; (2) it allows for the comparison of gene expression across multiple samples by normalizing to a reference gene, compensating for differences in sample tissue amounts; (3) the method provides a normalized expression ratio of the target gene in test samples compared to the control samples; and (4) it assumes that both target and reference genes have amplification efficiencies near 100% and within 5% of each other [28].

#### 2.5.2. VEGF Protein-Level Measurement

Plasma concentrations of VEGF were measured using commercially available ELISA kits obtained from “SunRed Biotechnology (Cat. no. 201-12-0051, Shanghai, China)”. This kit utilizes a double-antibody sandwich ELISA approach, recognized for its accuracy in quantifying VEGF levels in various samples. In summary, 40 µL of serum was introduced into a pre-coated well, followed by the addition of 10 µL of suPAR antibody and 50 µL of streptavidin-HRP. The mixture was then incubated at 37 °C for one hour. After removing the excess liquid, 50 µL of the chromogen solutions A and B were added, and the sample was incubated in the dark at 37 °C for 10 min. Subsequently, 50 µL of stop solution was added, changing color from blue to yellow. The intensity of the resulting color was positively correlated with VEGF concentration. The standards’ concentrations and corresponding optical densities (ODs) at 450 nm were utilized to establish a linear regression equation for determining the VEGF concentrations in the samples.

### 2.6. Data Analysis

The “Shapiro–Wilk normality” test was used to identify outliers in the data at a significant level of 0.05. Descriptive statistics were presented as means and standard deviations. Inferential statistics included the chi-square test for categorical variables. For quantitative variables, a “One-Way Analysis of Variance (ANOVA)” was utilized for parametric data, while the Kruskal–Wallis’s test was employed for non-parametric data. Spearman’s correlation coefficient was used for correlation analysis and presented as a correlation matrix plot.

The analysis of qRT-PCR outcomes employed the relative cycle threshold (Ct) method to compute gene expression levels via relative quantification using the 2^−[ΔΔCt] formula. Results more or less than one indicated the upregulation or downregulation of gene expression, respectively. The receiver operating characteristic (ROC) curve was employed to assess whether the levels of lncH19 and VEGF could distinguish between poor and good outcomes in DN patients compared to controls. The significance level was set at 0.05. Statistical analyses were conducted using the “Statistical Package for the Social Sciences (SPSS) software (IBM SPSS version 22.0)” and “GraphPad Prism version 8.0 for Windows (GraphPad Software, San Diego, CA, USA).” The software websites were last accessed on 4 May 2024.

## 3. Results

### 3.1. Bioinformatics Analysis Results

#### 3.1.1. miRNA -29b Selection

By searching the “Human microRNA Disease Database” for relationships between miRNAs and DN, over 20 miRNAs were associated with DM risk and complications. One of the most prominent miRNAs, miR-29, which was shown to target VEGF, has been known to mediate signaling pathways that can lead to the pathogenesis and development of DN. The results of the search are shown in (Table S1).

#### 3.1.2. In Silico Analysis for miRNA and lncRNA Target(s) Prediction and Pathway Enrichment Analysis

**Figure 2:**
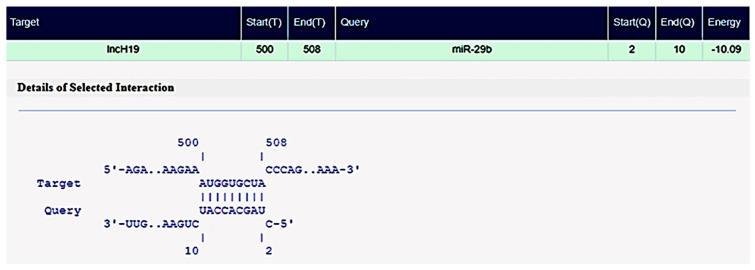
miR-29–lncH19 pairing showing the target binding sites. Source: starBase v2. database (http://starbase.sysu.edu.cn/, accessed 2 January 2024).

The starBase v2 database showed that miR-29b has several conserved binding sites on the non-coding sequences of lncH19, as shown in Figure 2. Although many studies have addressed the predicted functional roles of these ncRNAs separately, no reports have explored these two classes of ncRNAs simultaneously, particularly in patients with DN.

#### 3.1.3. MicroRNA-29b and lncH19 Sequence Analysis

**Figure 3:**
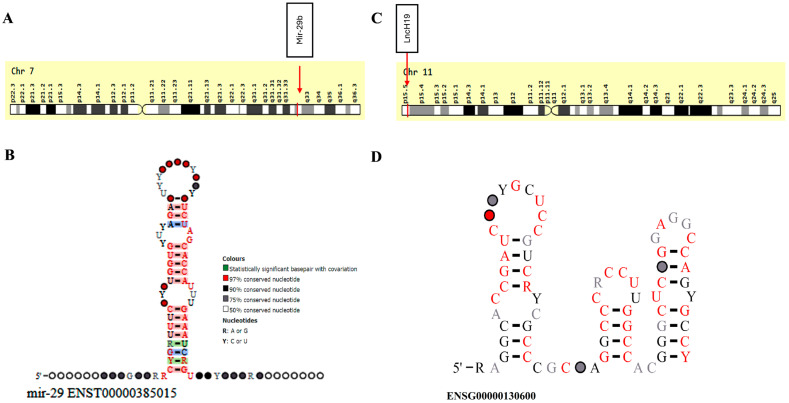
MicroRNA-29B and lncH19 gene structural analysis. (**A**) miRNA-29b chromosomal location and (**B**) miR-29b secondary structure, (**C**) lncH19 chromosomal location, and (**D**) lncH19 secondary structure. Source: https://useast.ensembl.org/Homo_sapiens/Gene/; last accessed 25 December 2024).

The analysis of miR-29b revealed:

- Genomic location: it is encoded by a single mature transcript located at the long arm of chromosome 7q32.3 (Chr. 7: 130,877,459–130,877,539) reverse strand, according to the GRCh38.p14 assembly (Figure 3A).
- Sequence characteristics: the mature miR-29b sequence is 22 nucleotides long, with the following sequence: 5′-UAGCACCAUUUGAAAUCAGUGUU-3′ [29].
- Secondary structure prediction: The pre-miR-29b forms a characteristic stem–loop structure (Figure 3B). The consensus and nucleotide sequence conservation of the gene family to which this microRNA belongs were illustrated.
- Conservation analysis: the miR-29b sequence is highly conserved across mammalian species, suggesting functional importance (Figure S1).

The analysis of lncH19 revealed:

- Genomic location: lncH19 is in an imprinted segment located on the short arm of chromosome 11p15.5 (Chr. 11: 1,995,165–2,004,552) reverse strand (Figure 3C).
- RNA length: the LncH19 gene codes for a 2.3 kb RNA product.
- Splice transcripts: lncH19 can generate up to 46 splice transcripts, with its resultant product functioning as a tumor suppressor protein.
- An analysis of the H19 RNA sequence using thermodynamic free-energy methods demonstrated the possibility of various secondary RNA structures, featuring 16 helices and different types of hairpin loops (Figure 3D).

#### 3.1.4. miR-29b, lncH19, and VEGFA Loop Interaction

**Figure 4:**
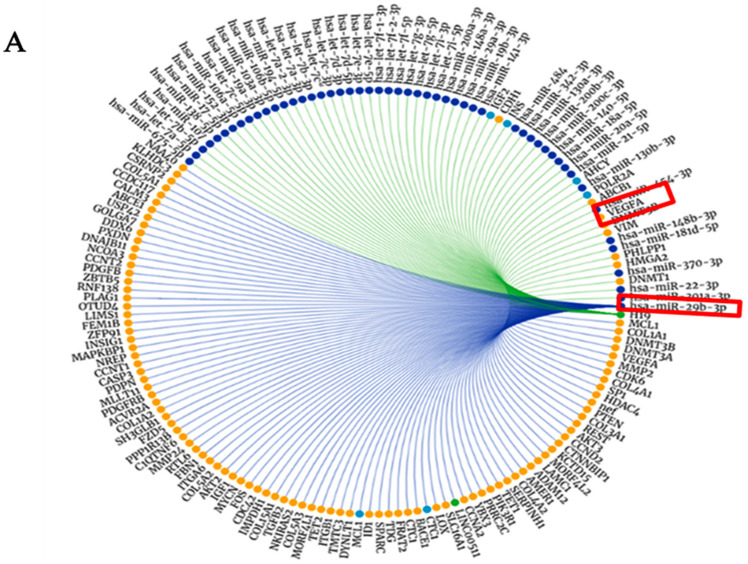

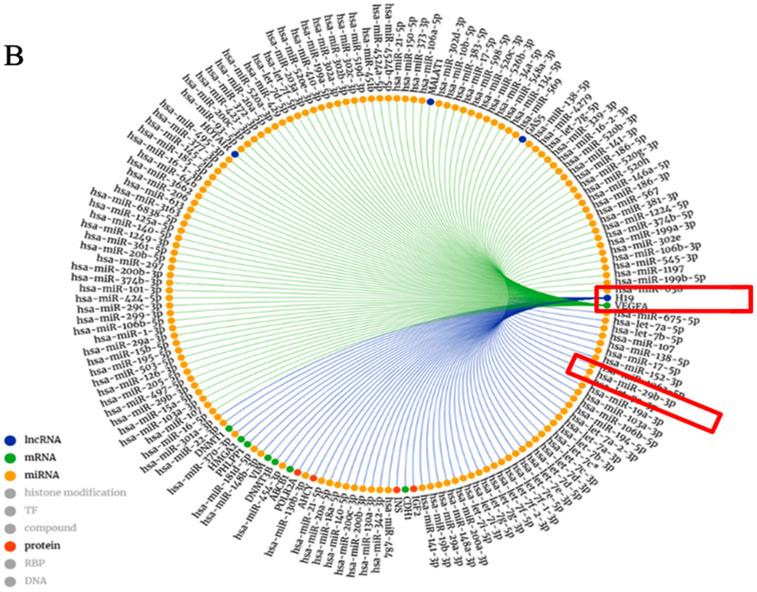
MiR-29b-3p, lncH19, and VEGF evidence of cross interactions. (**A**) miR-2b with VEGF; (**B**) lncH19 loop interaction with miR-29b and VEGF.

The RNAInter v3.0 database presented the top 100 interactors for miR-29b and lncH19, indicating the VEGFA as a common interactor between them, as illustrated in Figure 4.

#### 3.1.5. Target Genes and Pathway Analysis

**Figure 5:**
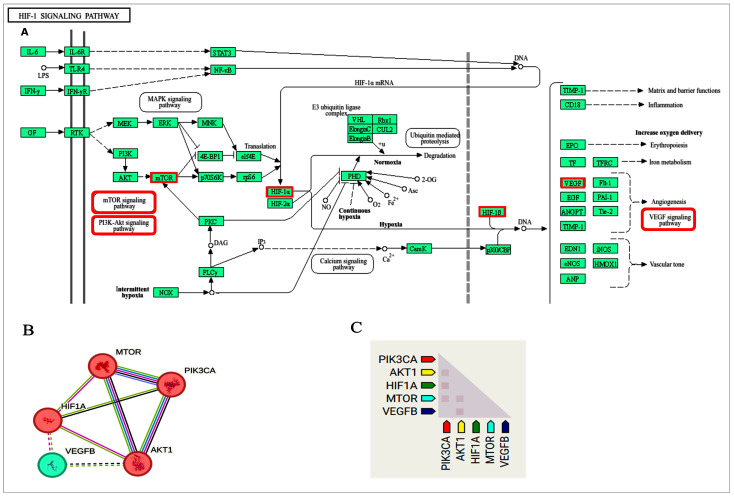
Interaction analysis for “PI3K/AKT/HIF-1” pathway. (**A**) Target pathways and genes (red boxes) in enriched KEGG pathway. The original pathway is adopted with permission (https://www.kegg.jp/pathway/map04066) (last accessed 20 November 2024) [23], (**B**) protein–protein interaction and clusters, and (**C**) coexpression analysis (Data sources: STRING database).

Clustering the functional annotation for the curated targeted genes and their enriched pathway analysis demonstrated that miR-29b-3p can target and, hence, regulate multiple molecular and signaling pathways involved in inflammatory and immunological biological processes. One of these pathways is the HIF-1 signaling pathway. When searching the KEGG database, the pathway was found to be initiated by the stimulation of PI3K/AKT/mTOR and concluded with the involvement of VEGFA, which is an essential marker for angiogenesis (Figure 5A). Performing protein–protein interaction (PPI) analysis revealed that the studied target genes are classified into two clusters with an average k-means clustering coefficient of 0.867 and highly significant interactions (*p*-value = 0.028) (Figure 5B). Confirming the coexpression data showed that PI3K plays a key role by recruiting AKT, which in turn activates VEGF. Additionally, PI3K is coexpressed with mTOR and HIF-1, which play a central role in oxidative stress. The overall coexpression score was 0.060, derived from GEO microarray expression data (Figure 5C).

### 3.2. The Baseline and Clinical Characteristics of the Investigated Groups

The age and sex of the participants in the control and patient groups were comparable, as indicated by the demographic characteristics presented in Table 2. The average age of the control group was 53, comparable to the patients’ age of 55. Also, both sexes were equally represented in all study groups. Significant differences were noted between the control and other groups regarding BMI (body mass index) and waist circumference. Furthermore, the duration of diabetes, family history of diabetes, and duration of receiving diabetic medications differed significantly between affected groups. Associated chronic diseases (hypertension, coronary artery disease, neuropathy, retinopathy, peripheral artery diseases, and coma occurrence) were increased within diseased groups, especially with increased renal affection. Most patients with macroalbuminuria lie in stage 4, while most of the microalbuminuric group lie in stages 1 and 2.

### 3.3. A Baseline Laboratory Investigation of Patients with Diabetic Nephropathy Among the Studied Groups

The results exhibited a statistically significant disparity among the groups regarding routine diabetic nephropathy laboratory biomarkers such as HB, PLT, TLC, FBS, lipid profile, liver enzymes, kidney function, and eGFR (Table 3).

### 3.4. Circulatory lncH19 and miR-29b Expression Levels

**Figure 6:**
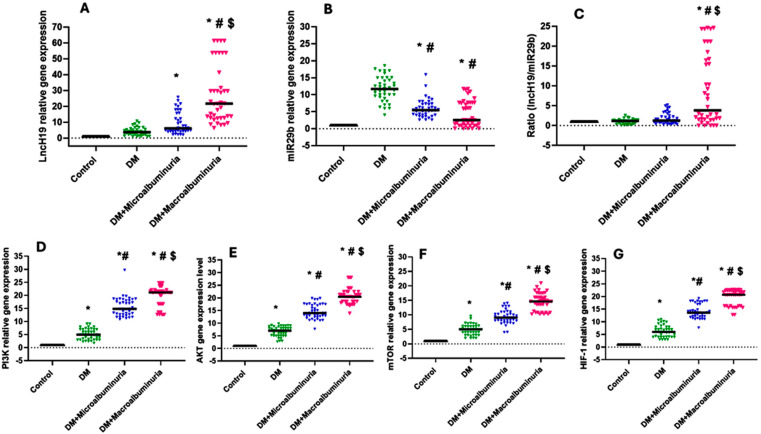
Gene expression among the four investigated groups, including the DM (green), DM with microalbuminuria (blue), and DM with macroalbuminuria (red) for lncH19 (panel **A**), miR-29b (panel **B**), their ratio (panel **C**) and target genes (i.e., panel **D**: PI3K, panel **E**: AKT, panel **F**: mTOR, and panel **G**: HIF-1). Data are presented as median and interquartile range; Kruskal–Wallis was employed to calculate the *p*-values, where * denotes a significant difference vs. the control group, # indicates a significant difference vs. the DM group, and $ indicates significant differences vs. the microalbuminuria group at *p* < 0.05.

The qRT-PCR method was employed to examine the relative expression levels of lncRNA H19 and miRNA29b and their target genes in patients with DN and healthy controls. Our investigation revealed that the relative expression levels of lncRNA H19 were notably increased in patients with macroalbuminuria compared to the control group and the DM group (Figure 6A). At the same time, the relative expression level of miR-29b significantly decreased in the DM and DM with albuminuria groups compared to the control group (Figure 6B). Concerning the lncRNA H19/miR-29b ratio, it shows a significantly higher value in blood samples obtained from DM with macroalbuminuria group compared to the controls, patients with DM, or DM with microalbuminuria group (*p* < 0.05, Figure 6C). The same occurs for PI3K, AKT, mTOR, and HIF-1 expression levels (Figure 6D–G).

### 3.5. Circulating Level of Serum VEGFA

**Figure 7:**
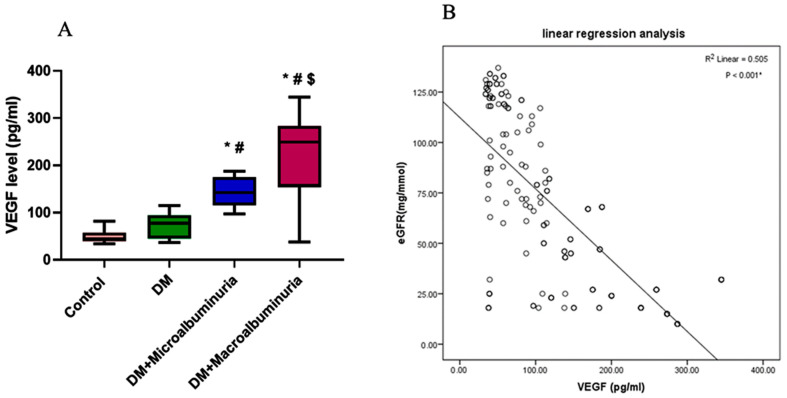
VEGFA protein analysis. (**A**) Circulating levels of serum VEGFA in the four studied groups. (**B**) The correlation between eGFR and the serum level of VEGFA. (Data were presented as median and interquartile range. Kruskal–Wallis tests were used to calculate the *p*-value where * denotes a statistically significant difference vs. the control group, # indicates a significant difference vs. the diabetic group, and $ indicates a significant difference vs. the microalbuminuria group at *p* < 0.05. R^2^ indicates the results of the Spearman correlation test, and the significance was determined at *p* < 0.05.

There was a significant increase in VEGFA in patients in the micro- and macroalbuminuric groups than in those in the DM or the control group (Figure 7A). Moreover, a significant negative correlation was found between serum levels of VEGF and eGFR (*p* < 0.001) (Figure 7B).

### 3.6. The Correlation Matrix for the Interrelationship Between lncH19 Expression Level and Other Study Variables

**Figure 8:**
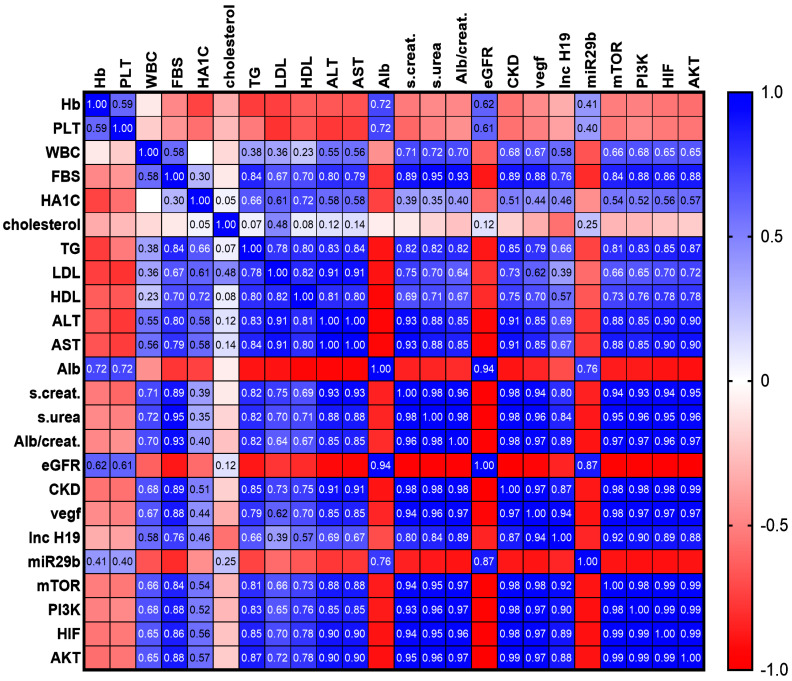
An illustration of the correlation matrix showing the connection between lncH19 level and various study variables. The blue color represents positive correlations, while the red color represents negative correlations. The value of R represents the strength of the association. Statistical significance was determined if *p*-value < 0.05.

The matrix showed a significant and robust positive correlation between the lncH19 level and VEGFA, mTOR, HIF-1, PI3K, and AKT (R^2^ = 0.94, 0.92, 0.89, 0.9, 0.88, respectively) and a weak negative correlation with miR-29b and eGFR (R^2^ = −1) (Figure 8).

### 3.7. Serum VEGF and Circulating lncH19 as a Prognostic Marker for Diabetic Nephropathy

**Figure 9:**
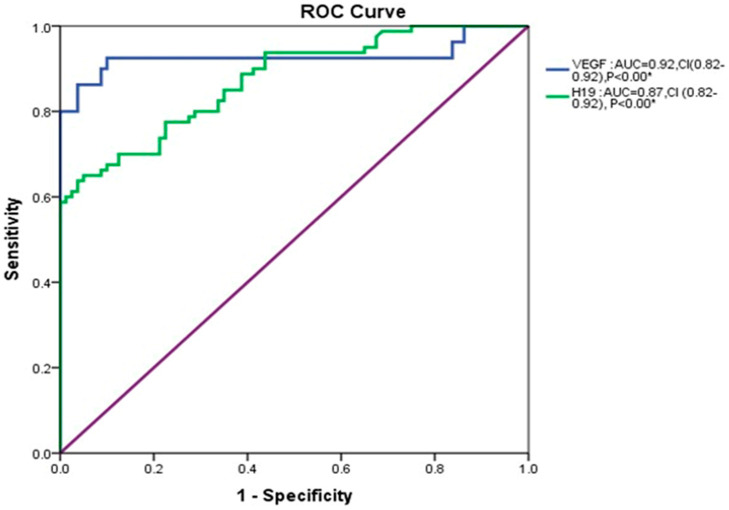
VEGF and lncH19 as an indicator for prognosis. ROC curve analysis for VEGF and lncH19 distinguishes patients with DN from those without DN. AUC: area under the curve, CI: Confidence Interval. * Statistical significance at *p*-value less than 0.05.

ROC analysis demonstrated that the serum VEGF level, which served as the cut-off value of 96.04 pg/mL, had a sensitivity of 96% and a specificity of 90% with an area under the curve (AUC) = 0.92 and a statistical significance difference less than 0.001. In contrast, lncH19 had a sensitivity of 92% and a specificity of 57%, with an area under the curve (AUC) = 0.87 and a significant difference of less than 0.001 (Figure 9).

## 4. Discussion

The negative consequences of diabetes continue to present a significant challenge, placing a heavy economic burden on nations. Exploring the bio-molecular mechanisms involved in diabetes may offer innovative solutions for diagnosing and treating this condition [30]. It is crucial to acknowledge the significance of lncRNAs in the development and advancement of DM/DN. This research showcases the dysregulation of lncH19, miR-29b, and VEGFA circulating levels in diabetic individuals with and without nephropathy compared to controls within a population from the “Middle East” region.

We have found that lncH19 is notably overexpressed in patients with DM, especially those with micro- and macroalbuminuria, compared to the control group. Prior research has indicated that lncH19 is commonly upregulated in renal disorders, including fibrosis, carcinoma, and cellular damage [31,32]. According to the study conducted by Wang et al., it was observed that the levels of lncH19 were elevated in renal cell carcinoma and showed a significant correlation with tumor stage and metastasis. Furthermore, their research findings suggested that a decrease in lncH19 expression resulted in a decline in the proliferation, migration, and invasion abilities of the cancer cells. These results propose that lncH19 has the potential to be utilized as a biomarker or therapeutic target for the treatment of renal cancer [33].

Fan et al. concurred with our findings by stating that the levels of lncH19 expression were higher in individuals with diabetic kidney disease compared to diabetic patients without nephropathy. This indicates that lncH19 could be a promising biomarker for diabetic kidney disease [31]. On the contrary, Xu et al. discovered that the expression of lncH19 was reduced in blood samples taken from children with nephrotic syndrome. They also found that lncH19 played a role in regulating the expression of the “AarF Domain-Containing Protein Kinase 4; *ADCK4*” gene, which is associated with nephrotic syndrome. However, these findings contradict our results and the findings of other studies that have reported elevated levels of lncH19 in kidney pathologies [34].

Additionally, our research revealed a significant discovery compared to the earlier studies: an upregulation in circulating lncH19. This increase in expression was not only linked to declining kidney function, but also correlated with various other parameters such as miR-29b, VEGFA, and other proliferating pathways and metabolic regulators such as PI3K/AKT/mTOR/HIF-1, which could potentially explain the pathogenesis of diabetic complications. MiR-29b was among the initial miRNAs that exhibited dysregulation in retinal cells and diabetic rats subjected to elevated glucose levels. This observation implies their potential involvement in the progression of DM [35].

Our study revealed that miR-29b is downregulated significantly in the DM with micro- and macroalbuminuria groups compared to the control group. This was consistent with an in vivo study that demonstrated that miR-29b plays a role in controlling glucose levels and insulin release by targeting multiple genes, thus impacting the development of diabetes [36].

We found that the serum level of VEGFA increased significantly in DM with micro- and macroalbuminuria groups compared to the control group. The VEGF glycoprotein family demonstrates a strong ability to promote the division and growth of vascular endothelial cells, along with enhancing capillary permeability. This family includes VEGFA-D and the placenta growth factor. VEGFA has shown notable efficacy with vascular endothelial cells [37]. It serves as a direct target gene for numerous miRNAs. For example, miR-9 hinders the development of new blood vessels in the retina and the formation of tubules in diabetic retinitis, as well as inducing apoptosis in retinal microvascular endothelial cells by explicitly targeting VEGFA [38]. MiR-15a-5p acts as a controller of VEGFA mRNA and has a crucial role in regulating inflammation and fibrosis in peritoneal mesothelial cells [39]. These findings prompted us to conduct a more extensive examination into the potential role of H19/miR-29b/VEGFA in the inflammatory response among individuals diagnosed with DN.

The results showed that, compared with the control group, the serum levels of PI3K, AKT, mTOR, and HIF-1α were significantly elevated in diabetic patients with micro- and macroalbuminuria than in controls. A correlation analysis showed that PI3K, AKT, mTOR, HIF-1α, and VEGF were positively correlated with lncH19. Our findings were consistent with the research conducted by Isoe et al., indicating that under hyperglycemic conditions, there was a notable increase in the protein and mRNA levels of HIF-1α in mesangial cells [40]. Under typical oxygen conditions, the transcriptional activity of HIF-1α could be enhanced by adjusting the mitogen-activated protein kinase due to advanced glycation end-products. Moreover, Tang et al. verified in both in vivo and in vitro experiments that HIF-1α stimulates renal fibrosis by activating angiotensin II [41]. Shao et al. found the same results, where they proposed that abnormal angiogenesis is responsible for the development of immature blood vessels, leading to renal fibrosis and ultimately causing the decline of glomerular function in DKD. The upregulation of VEGF expression and downregulation of endothelial nitric oxide synthase expression contribute to the abnormal formation of blood vessels in DKD. The overexpression of VEGF enhances the activation of the PI3K/AKT signaling pathway, leading to the phosphorylation of endothelial nitric oxide synthase and subsequent angiogenesis, thereby facilitating the progression of DKD [42].

We have discovered a new pathogenic connection involving lncH19, miR-29b, and VEGFA in DN. The inhibition of lncH19 could potentially reduce inflammation in endothelial cells through the upregulation of miR-29b and the suppression of VEGFA expression.

Interestingly, our study explored that VEGFA and lncH19 have a high prognostic value (AUC = 0.92 and AUC = 0.87, respectively), distinguishing diabetic patients from those with poor outcomes as DN. This result is in agreement with Alfaifi et al., who reported that they had been demonstrated to serve as important diagnostic/prognostic biomarkers in diabetes [43].

## 5. Conclusions

Our research revealed that circulating levels of lncH19 were higher in patients with DN, and these elevated levels may be linked to various inflammatory and angiogenic markers. Additionally, lncH19/miR-29b could hold significant clinical value in understanding the development of DN and other diabetes-related complications. Nevertheless, further comprehensive studies are necessary to fully comprehend the involvement of lncH19 in the molecular pathways associated with DN, as well as explore the potential therapeutic effects of inhibiting lncH19 and the interaction with VEGFA in DN patients. These data highlight the importance of gene interactions in the onset and progression of the disease. Information on the expression patterns of lncH19 and miR-29b could be crucial for the diagnosis, prognosis, and management of DN cases.

## Figures and Tables

**Table 1:** The primer sequences of the studied genes in the present study.

Gene	Primers Sequences	Accession Number	Annealing Temperature
*PI3K*	Forward: 5′-GAAGCACCTGAATAGGCAAGTCG-3′ Reverse: 5′-GAGCATCCATGAAATCTGGTCGC-3′	NM_006218.4	60 °C
*AKT*	Forward: 5′-TATAAGGGGAGGTGGGGTCA-3′ Reverse: 5′-CCTACTCTATCAGGCTGCCC-3′	NM_001382433.1	60 °C
*mTOR*	Forward: 5′-AGCATCGGATGCTTAGGAGTGG-3′ Reverse: 5′-CAGCCAGTCATCTTTGGAGACC-3′	NM_004958.4	60 °C
*HIF-1 alpha*	Forward: 5′-TATGAGCCAGAAGAACTTTTAGGC-3′ Reverse: 5′-CACCTCTTTTGGCAAGCATCCTG-3′	NM_001530.4	60 °C
*miR-29b*	Forward: 5′-TCAGGAAGCTGGTTTCATATGGT-3′ Reverse: 5′-CCCCCAAGAACACTGATTTCAA-3′	MIMAT0004514	60 °C
*LncH19*	Forward: 5′-CACTGGCCTCCAGAGCCCGT-3′ Reverse: 5′-CGTCTTGGCCTTCGGCAGCTG-3′	NR_002196	60 °C

**Table 2:** Demographic and clinical data of study participants.

Variables		(Control)	DM	DM with Microalbuminuria	DM with Macroalbuminuria	*p*-Value
		*n* = 40	*n* = 40	*n* = 40	*n* = 40	
		N (%)	N (%)	N (%)	N (%)	
Sex	Male	20 (11.3%)	20 (12.5%)	20 (12.5%)	20 (12.5%)	0.96
	Female	22 (138%)	20 (12.5%)	20 (12.5%)	20 (12.5%)	
Age	Mean ± SD	53 ± 10	53.8 ± 10.5	56 ± 10.8	57 ± 10.3	0.6
BMI	Mean ± SD	24.48 ± 2.1	32.07 ± 4.4	31.3 ± 4.7	32.06 ± 5.016	<0.001 *
Waist circumference	Mean ± SD	83.8 ± 7.1	115.8 ± 17.6	104.5 ± 13.48	106.6 ± 7.49	<0.001 *
DM duration	Mean ± SD		9.17 ± 6.9	12.1 ± 8.9	23.6 ± 8.7	<0.001 *
Duration of treatment	Mean ± SD		9.15 ± 6.9	12.1 ± 8.9	23.6 ± 8.7	<0.001 *
Family history of diabetes	Yes	15 (9.4%)	27 (16.9%)	36 (22.5%)	30 (18.8%)	<0.001 *
	No	25 (15.6%)	13 (8.1%)	4 (2.5%)	10 (6.3%)	
Smoking	Yes	15 (9.4)	9 (5.6%)	8 (5%)	11 (6.9%)	0.362
	No	25 (15.6)	30 (18.8%)	31(20%)	29 (18.8%)	
Hypertension	Yes	0 (0%)	13 (8.1%)	19 (11.9%)	20 (12.5%)	<0.001 *
	No	40 (25%)	27 (16.9%)	21 (13.1%)	20 (12.5%)	
Coronary artery disease	Yes	0 (0%)	5 (3.1%)	6 (3.8%)	13 (8.1%)	<0.001*
	No	40 (25%)	35 (21.9%)	34 (21.3%)	27 (16.9%)	
Peripheral artery disease	Yes	0 (0%)	4 (2.5%)	4 (2.5%)	13 (8.1%)	<0.001 *
	No	40 (25%)	36 (22.5%)	36 (22.5%)	27 (16.9%)	
Diabetic retinopathy	Yes	0 (0%)	3 (1.9%)	9 (5.6%)	25 (15.6%)	<0.001 *
	No	40 (25%)	37 (23.1%)	31 (19.4%)	15 (9.4%)	
Diabetic neuropathy	Yes	0 (0%)	11 (6.9%)	14 (8.8%)	40 (25%)	<0.001 *
	No	40 (25%)	29 (18.1%)	26 (16.3%)	0 (0%)	
Coma	Yes	0 (0%)	14 (8.8%)	8 (5%)	30 (18.8%)	<0.001 *
	No	40 (25%)	26 (16.3%)	32 (20%)	10 (6.3%)	
Type of treatment	Oral	0 (0%)	28 (17.5%)	21 (13.5%)	0 (0%)	<0.001 *
	Insulin	40 (25%)	11 (7.5%)	19 (11.9%)	40 (25%)	
	No	40 (25%)	40 (25%)	35 (21.9%)	35 (21.9%)	
CKD stage	Stage I	0 (0%)	15 (9.4%)	0 (0%)	0 (0%)	<0.001 *
	Stage II	0 (0%)	23 (14.4%)	12 (7.5%)	0 (0%)	
	Stage IIIa	0 (0%)	1 (0.6%)	13 (8.1%)	0 (0%)	
	Stage IIIb	0 (0%)	1 (0.6%)	3 (1.9%)	5 (3.1%)	
	Stage 4	0 (0%)	0 (0%)	12 (7.5%)	30 (18.8%)	
	Stage 5	0 (0%)	0 (0%)	0 (0%)	5 (3.1%)	

Data are displayed as frequency (N), percentage (%), or mean ± standard deviation (SD); one-way ANOVA and Chi-square were employed to calculate the *p*-values. * Significant at *p* < 0.05. “DM: Diabetes Mellitus, BMI: Body Mass Index, CKD: Chronic kidney disease”.

**Table 3:** Baseline laboratory results of studied diabetic nephropathy patients and controls.

Variables	(Control)	DM	DM with Microalbuminuria	DM with Macroalbuminuria	*p*-Value
	n = 40	n = 40	n = 40	n = 40	
HB (g/dL)	13.3 ± 0.7	12.3 ± 1.6 *	12.7 ± 1.2	12.5 ± 05 *	<0.001
PLT (×10^3^/µL)	285.3 ± 60.9	205.9 ± 30.9 *	232.7 ± 73.3 *#	247.5 ± 81.6	<0.001
TLC (×10^3^/µL)	7322.5 ± 1428.7	5999.8 ± 939.3 *	8202.5 ± 1772.4 *#	8275 ± 1256.5 *#	<0.001
FBS (mg/dL)	90.6 ± 7.1	138.6 ± 61.4 *	127.9 ± 48.4	237.6 ± 107.8 *#$	<0.001
HA1c	5.1 ± 0.3	7.5 ± 1 *	7.7 ± 3.2 *	8.3 ± 1.6 *	<0.001
Cholesterol (mg/dL)	167 ± 24.7	202 ± 33.9 *	158 ± 53.6 #	173 ± 47.1 #	<0.001
Triglycerides (mg/dL)	83 ± 23.8	138 ±39.5 *	125 ± 26.8 *	170 ± 55 *#$	<0.001
LDL (mg/dL)	134 ± 33.2	130 ± 35.4 *	94 ± 44.6 *#	119 ±55.8 *$	<0.001
HDL (mg/dL)	64 ± 9	43 ± 10 *	44.5 ± 12.6 *	39.3 ±5.5 *$	<0.001
ALT (U/L)	25 ± 6.5	29 ±10.5 *	27 ± 10.8 *	43 ± 19.9 *#$	<0.001
AST (U/L)	26 ± 7.1	26 ± 8.7 *	28 ±11.3 *	48 ± 27.8 *#$	<0.001
Albumin (mg/dL)	4.5 ± 0.4	4.4 ± 0.5	4.3 ± 0.4	3.5 ± 0.6	<0.001
Serum Creatinine (mg/dL)	0.6 ± 0.06	0.9 ± 0.2	1.6 ± 0.7 *	3.3 ± 1.3 *#$	<0.001
Blood Urea (mg/dL)	13 ± 3.5	21 ± 10.8	45 ± 12.8 *	81 ±28.3 *#$	<0.001
Urinary creatinine albumin ratio (mg/g)	11 ± 2.6	19.7 ± 12.2	99.5 ± 57.1 *#	452 ± 78 *#$	<0.001
eGFR (mg/mmoL)	11.2 ± 2	12.1 ± 2.3 *	110 ± 20 *#	560 ± 65 *#$	<0.001

Data are represented as mean ± SD. One-way ANOVA was employed to calculate the *p*-values. Where (* indicates a level of significance vs. the control group, # indicates a level of significance vs. the diabetic group, and $ indicates a level of significance vs. the microalbuminuria group at *p* < 0.05. “HB: hemoglobin, PLT: platelet, TLC: total leucocytic count, FBS: fasting blood sugar, HA1c: glycated hemoglobin, LDL: low-density lipoproteins, HDL: high-density lipoproteins, ALT: alanine aminotransferase, AST: aspartate aminotransferase, eGFR: estimated glomerular filtration rate, VEGFA: vascular endothelial growth factor alpha, lncH19: long non-coding H19, miR-29b, HIF: hypoxia-inducible factor”.

## Data Availability

The original contributions presented in the study are included in the article/Supplementary Material; further inquiries can be directed to the corresponding author.

## Supplementary Materials

The following supporting information can be downloaded at: https://www.mdpi.com/article/10.3390/cimb47010020/s1.
