# Supplementary material for: The Association of Cell-Free LncH19 and miR-29b Expression with the PI3K/AKT/HIF-1/VEGF Pathway in Patients with Diabetic Nephropathy: In Silico Prediction and Clinical Validation
Source: Curr Issues Mol Biol. 2024 Dec 31;47(1):20. doi: 10.3390/cimb47010020 (PMC11764133; doi:10.3390/cimb47010020)
Supplement: Supplementary file 1 [file cimb-47-00020-s001.zip › cimb-3355413-supplementary.pdf]

Table S1: The "Human microRNA Disease Database" search results. miRNA name: hsa-mir-29b

Category	Disease_name	PMID	DOID	ICD10CM	MESH	OMIM	Description	Causality
genetics_knock down_promote	Carcinoma, Renal Cell	26823729	DOID:4450		D002292	144700	Inhibition of miR-29b expression could promote apo	YES
genetics_overexpression_promot	Hearing Loss	27635430		H91.93	D034381		miR-29b overexpression induces cochlear hair cell a	YES
genetics_overexpression_suppre	Prostatic Neoplasms	25784815	DOID:1028	C51	D011471	176807	forced expression of miR-29b inhibited cell prolifera	YES
genetics_overexpression_suppre	Liver Cirrhosis	27273381	DOID:5082	K74	D008103	215600	miR-29b overexpression inhibited proliferation of LX	YES
genetics_overexpression_suppre	Wound Healing	27477081			D014945		Importantly, local delivery of miR-29b lentiviral parti	YES
genetics_overexpression_suppre	Corneal Dystrophies, Hereditary	27490049	DOID:1231	H18.53	D003317	121900	Overexpression of miR-29b decreased ECM protein	YES
lncRNA target	Diabetic Retinopathy	28246353	DOID:8947	E10-11.31	D003930		Long non-coding RNA MIAT acts as a biomarker in di	YES
target gene	Carcinoma, squamous cell of head and neck	24091622	DOID:5520	C76.0	C535575		Downregulation of miR-29s was a frequent event in	YES
target gene	Colorectal Neoplasms	24913975	DOID:0080	C19	D015179	114500	MiR-29b downregulates canonical Wnt signaling by	YES
target gene	Colorectal Neoplasms	25032858	DOID:0080	C19	D015179	114500	miR-29b suppresses tumor growth and metastasis ir	YES
target gene	Diabetes Mellitus	25062042	DOID:9351	E10-E14	D003920	222100	insulin and mTORC1 regulate cardiac miR-29-MCL-1	YES
target gene	Arbovirus Infections	25234643	DOID:934	A94	D001102		Lentivirus-mediated Bos taurus bta-miR-29b overex	YES
target gene	Liver Cirrhosis	25356754		K74	D008103		microRNA-29b prevents liver fibrosis by attenuating	YES
target gene	Colorectal Neoplasms	25592039	DOID:0080	C19	D015179	114500	the potential signaling pathway,IFN-γ/IRF1/miR-29b,	YES
target gene	Breast Neoplasms	25622979	DOID:1612	C50	D001943	114480	The distinct modulations of the NF-κB - miR-29b - p5	YES
target gene	Nasopharyngeal carcinoma	25786138	DOID:9261	C11	C538339	607107	miR-29a/b enhances cell migration and invasion in n	YES
target gene	Neoplasms	26096783		C80.1	D009369		Tumour-suppressive microRNA-29s directly regulate	YES
target gene	Glioblastoma	26155940			D005909		miR-29b attenuates tumorigenicity and stemness m	YES
target gene	tongue squamous cell carcinoma	26885901	DOID:0050	C02.9			Relationships between microRNA expressions and p	YES
target gene	Breast Neoplasms	28365400	DOID:1612	C50	D001943	114480	MiRNA-29b suppresses tumor growth through simul	YES
target gene	Breast Neoplasms	29256222	DOID:1612	C50	D001943	114480	MiRNA-29b can inhibit the proliferation, invasion an	YES
target gene	Neuroblastoma	29399057	DOID:769	C74.90	D009447	256700	MicroRNA-29b alleviates oxygen and glucose depriv	YES
target gene	Renal fibrosis	29568897	DOID:0050	N26.9			miR-29b regulates Ang II-induced EMT of rat renal tu	YES
target gene	Hypertension, Pulmonary	29662889	DOID:6432	I27.20	D006976	PS178600	Effect of miR-29b on the Proliferation and Apoptosis	YES
target gene	Inflammation	29665646			D007249		miR-29b could regulate LPS-induced endothelial cell	YES
target gene	Depressive Disorder, Major	30369596	DOID:1470	F32	D003865	608520	The miR-29b-3p/GRM4 pathway acts as a critical me	YES
therapeutic target	Cardiovascular Diseases	24569834			D002318		In conclusion, miR-29b plays a protective role in Ang	YES
therapeutic target	Atherosclerosis	25131924	DOID:1936	I70	D050197	108725	The effect of miR-29b on endothelial permeability ar	YES
therapeutic target	Oral squamous cell carcinoma	25435433	DOID:0050866				MiR-29b acts as an oncomir, promoting cell migratic	YES
therapeutic target	Lung Neoplasms	26063204	DOID:1324	C34.1-.3	D008175		Taken together, our results demonstrate that miR-2	YES
therapeutic target	Brain Ischemia	26126866	DOID:2316	I67.82	D002545		MicroRNA-29b is a therapeutic target in cerebral iscl	YES
therapeutic target	Sarcoma, Ewing	26393798	DOID:3369		D012512	612219	These findings suggests that the up-regulation of c-N	YES
therapeutic target	Multiple Myeloma	27196750	DOID:9538		D009101	254500	miR-29b-based epi-therapeutic approaches in the tr	YES
therapeutic target	Glioblastoma	29176935			D005909		miR-29b may serve as a putative therapeutic molecu	YES

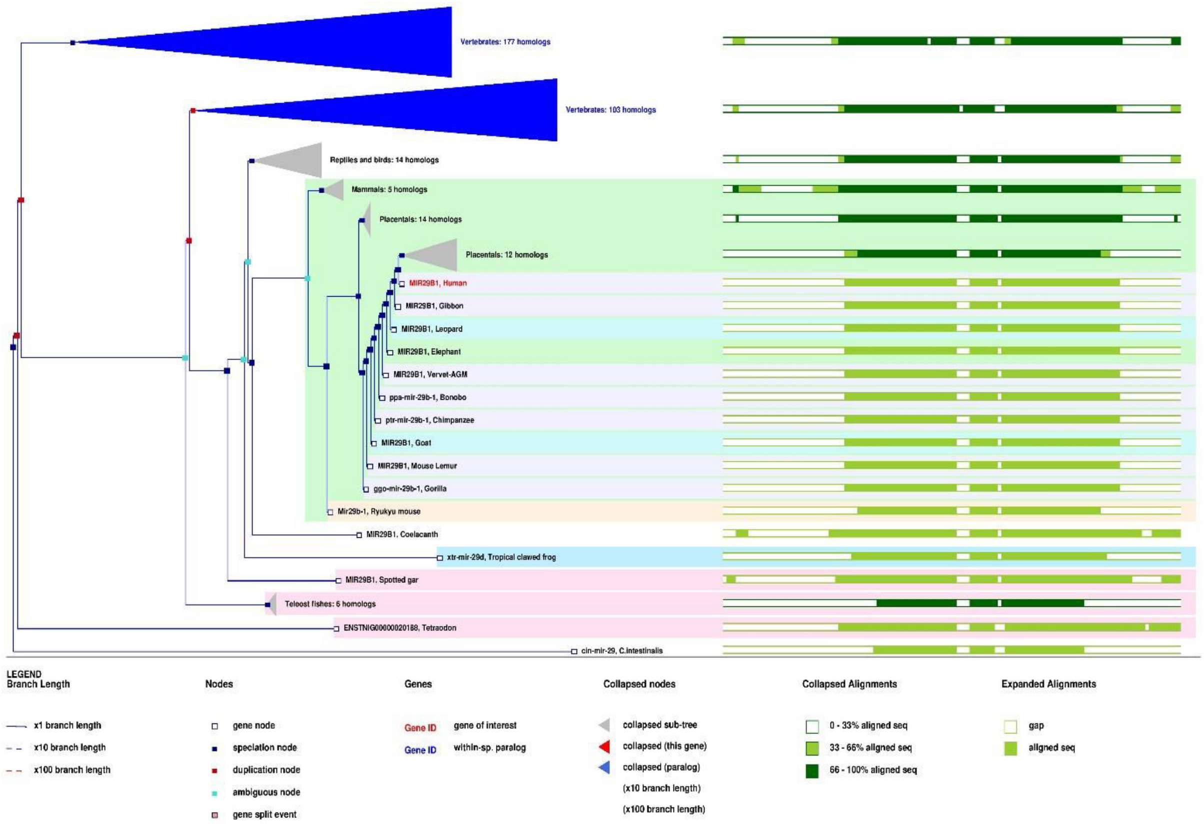

Figure S1: MicroRNA-29B (RF00074) shows conservation across vertebrates (data source: http://asia.ensembl.org/ (accessed 26 December 2024)).
